# Pitfalls in Portacath location using the landmark technique: case report

**DOI:** 10.1186/1477-7800-4-13

**Published:** 2007-06-05

**Authors:** Susannah M Wyles, Garry Browne, Gerald PH Gui

**Affiliations:** 1 Academic Surgery (Breast Unit) and, Royal Marsden NHS Foundation Trust, London, UK; 2 Department of Anaesthetics, Royal Marsden NHS Foundation Trust, London, UK

## Abstract

A 34 year old woman diagnosed with breast cancer and liver metastases underwent a left subclavian Portacath insertion. During the procedure, the clinical features and the findings of intra-operative investigations provided conflicting evidence of the catheter position. This report highlights the potential difficulties in establishing long-term central venous access, the limitations of common investigations and safety issues relating to the process of subclavian line insertion.

## Background

Portacaths (Bard Medical Division, Georgia, USA) are routinely used for central venous access in patients with poor peripheral veins who require chemotherapy, either in the adjuvant or metastatic disease setting. The technique provides a permanent, closed venous system with easy vascular access for drug delivery and blood draw that is more discrete and convenient than Hickmann lines and peripherally inserted central catheters (PICC), where part of the system lies ex-vivo. We present a case report that highlights potential difficulties in identifying the catheter position and consider the limitations of common methods of confirming the catheter location.

## Case presentation

A woman was aged 34 years when she presented with a T2 G2 N0 right breast cancer that was ER negative, PgR negative and HER-2 3+. She was otherwise well with no family history of cancer. She was started on 5 fluorouracil, epirubicin and cyclophosphamide (FEC) chemotherapy with gosarelin (Zoladex) and recombinant human granulocyte-colony stimulating factor, G-CSF, (Filgrastim) support. Prior to starting trastuzumab (Herceptin), a MUGA scan was performed showing a normal resting gated left ventricular ejection fraction of 57% and a normal size left ventricle. As she described increasing shortness of breath over the proceeding 6 months, a CT scan was done which showed no abnormality in the lung parenchyma but several scattered metastases were identified within the liver varying in size up to 3.5 cm with the largest lesion in segment 6.

With minimal response to chemotherapy, she proceeded to transarterial hepatic chemo-embolisation with mitomycin C/gemcitabine and converted to docetaxel (Taxotere) after 2 cycles of FEC. She was referred for Portocath insertion after 3 cycles of 2 weekly docetaxel for her ongoing taxane/trastuzumab chemotherapy. Her haemoglobin fell to 8.5 g/dL following chemotherapy at the time of Portocath insertion.

A left subclavian approach using the landmark technique was chosen. The vein was cannulated at first pass. The colour of the aspirated blood was bright red and there was brisk, pulsatile backflow through the needle. As the guide wire appeared to be appropriately placed in the right atrium on the image intensifier, the procedure continued with dilation of the vein and insertion of the catheter. The per-operative catheter position was indeterminate on image intensifier screening. The patient was being ventilated with 100% oxygen (FiO_2 _= 1). A catheter blood sample revealed a PO2 of 16.7 kPa, PCO2 of 6.45 with a PH of 7.28. As the PO2 could still be compatible with an arterial sample, Ultravist 300 (Lopromide) (Schering, West Sussex, UK) was injected down the catheter but the contrast dissipated so rapidly that the screening images were unhelpful in distinguishing arterial from venous catheterisation. Pulsatile blood flow continued despite holding the catheter 20cm above the patient's chest. Manometry was therefore used to provide objective pressure readings. The initial reading of 55/6 mmHg was interpreted to be in the right ventricle and drawing the catheter back with screening measured the superior vena cava pressure to be 22/4 mmHg. The arterial blood pressure at the time was 105/70 mmHg. We were thus finally convinced of the true catheter position based on the anatomical images and the physiological pressure readings. Appropriate catheter position was confirmed on the post-procedure chest X-ray.

## Discussion

Difficulty in identifying the catheter position occurs occasionally and this report illustrates commonly used procedures to establish the true location of the catheter. This case demonstrates that none of these methods are individually reliable because of variation in anatomy or altered physiology from disease or its treatment. In our case, as the procedure was performed under a general anaesthetic in the operating theatre, we had full control of the situation with readily available equipment and monitoring. We were able to confirm the catheter position without unnecessary withdrawal and re-cannulation of the vessel, thus avoiding the risk of inadvertent iatrogenic injuries.

In breast cancer, anthracycline chemotherapy and trastuzumab are frequently used and in combination can predispose to cardiac failure. The presence of significant liver metastases could increase the venous pressure and anaemia further predisposes to a hyperdynamic circulation. An increase in the number of requests for long term venous catherisation can be anticipated in patients who are on chemotherapy and biological treatment such as trastuzumab. Procedures to establish long term central venous catheterisation may be carried out in a variety of hospital locations depending on local practice including wards and radiology departments where access to monitoring may be less available. When difficulties are encountered, the complications can be distressing to patients. Operating theatres and radiological vascular intervention suites are well equipped for this purpose.

Hind et al performed a meta-analysis reviewing the use of ultrasound for central venous catheter placement and advocated the routine use of two dimensional ultrasonography [1]. The case for the use of ultrasound was stronger for internal jugular vein catheterisation compared with cannulation of the subclavian or femoral veins. The National Institute of Clinical Excellence guidance also advocates the use of ultrasound for internal jugular vein placement [2]. The overall safety of permanent venous devices and acceptable long-term complications was reviewed in a retrospective study of 1422 procedures by Sariego et al [3]. In our case, access to the subclavian vein was not the issue but uncertainty about the position of the catheter once line access had been established was the cause of consternation. We usually use the landmark technique for subclavian vein cannulation but have access to per-operative ultrasound in the operating theatre should there be difficulty in obtaining venous access which was not the case in this patient.

Crozier et al (2006) reviewed the landmark technique with experienced operators. The complication rates were low, affecting only 14 of 201 patients (7%) over a five year period [4]. Gopal et al (2006) evaluated the success and complication rate of nurse-led subclavian central venous catheter insertion using the landmark technique over a six month period [5]. Complications occurred in 48 out of 348 lines (14%), including catheter malposition, arterial puncture and pneumothorax, with an overall procedure failure rate of 1%. While nurse-led subclavian vein cannulation using the landmark technique was both safe and effective, the complication rates were twice that of an experienced medical operator.

Gualtieri et al reported the results of a randomized study in 1996 comparing subclavian venous catheterization by operators of variable experience [6]. They compared the success rate of using the landmark technique with ultrasound guidance, and found that ultrasound guidance improved the success rate of catheterization by less experienced operators. Ultrasound facility should be routinely available during the cannulation procedure at least as an adjunct to line placement.

## Conclusion

A complete understanding of the anatomy and physiology is an essential part of safe line placement. The placement of in-dwelling central venous catheters should be by dedicated teams within the hospital. Appropriate monitoring should be available for all patients by a trained clinician such as an experienced anaesthetist, separate from the practitioner inserting the catheter. Whilst there is an increasing tendency for central line placement to be devolved to nurse practitioners, we urge caution about the delegation of such responsibility without rigorous protocols on action in the event of difficulty.

## Abbreviations

ER (oestrogen receptor)

Her-2 (human epidermal growth factor receptor-2)

PR (progesterone receptor)

MUGA (multiple gated acquisition scan)

## Competing interests

The author(s) declare that they have no competing interests.

## Authors' contributions

SW performed the literature review and prepared the manuscript.

GB helped with the acquisition and interpretation of data and contributed to the final manuscript.

GG identified the educational value of the report, reviewed the literature and prepared the manuscript.

All authors read and approved the final manuscript.
